# Supplementary figures and images for: Bidirectional gated recurrent unit network model can generate future visual field with variable number of input elements
Source: PLoS One. 2024 Aug 27;19(8):e0307498. doi: 10.1371/journal.pone.0307498 (PMC11349096; doi:10.1371/journal.pone.0307498)

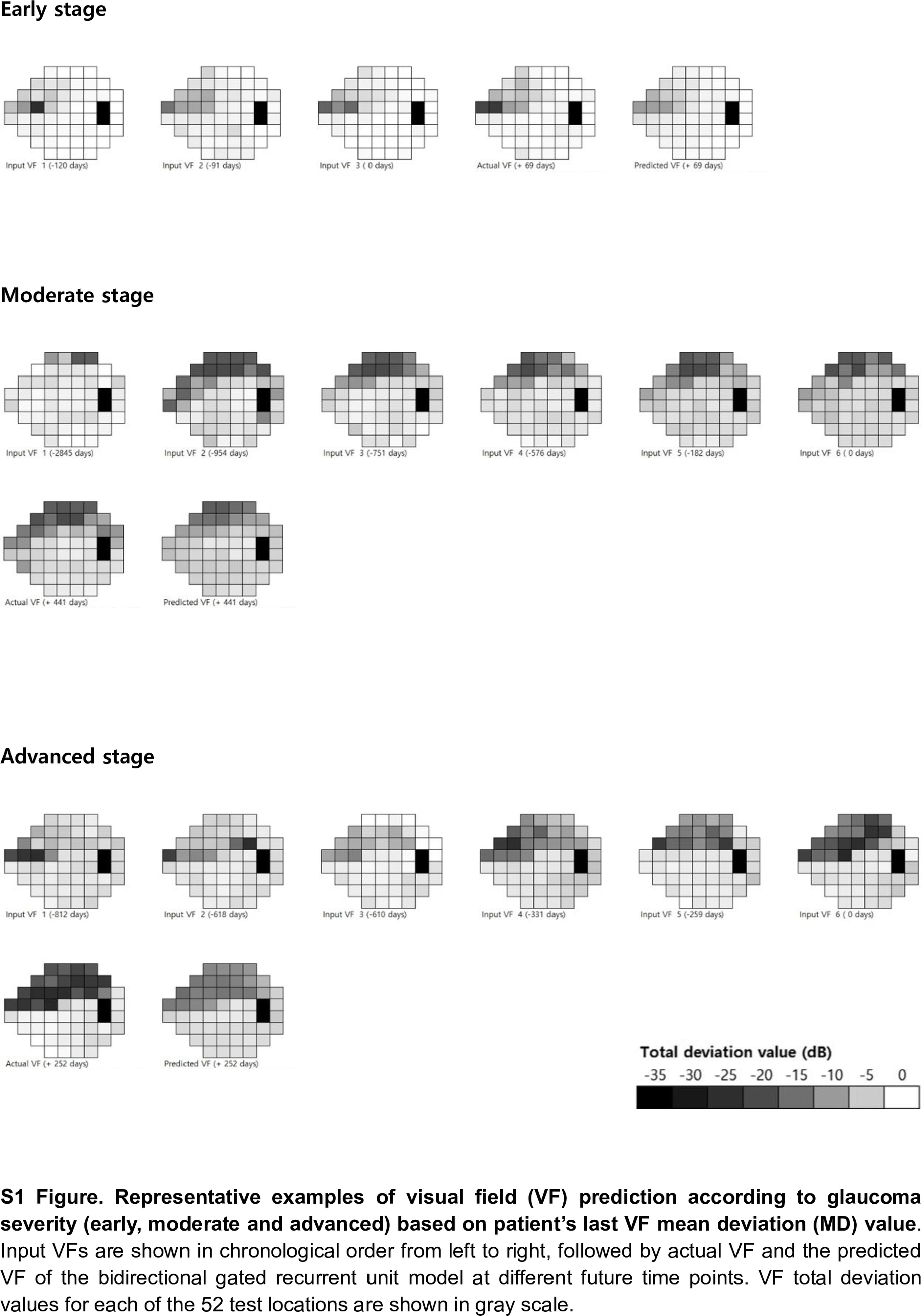

Supplement: S1 Fig — Input VFs are shown in chronological order from left to right, followed by actual VF and the predicted VF of the bidirectional gated recurrent unit model at different future time points. VF total deviation values for each of the 52 test locations are shown in gray scale. (TIF) [file pone.0307498.s001.tif]

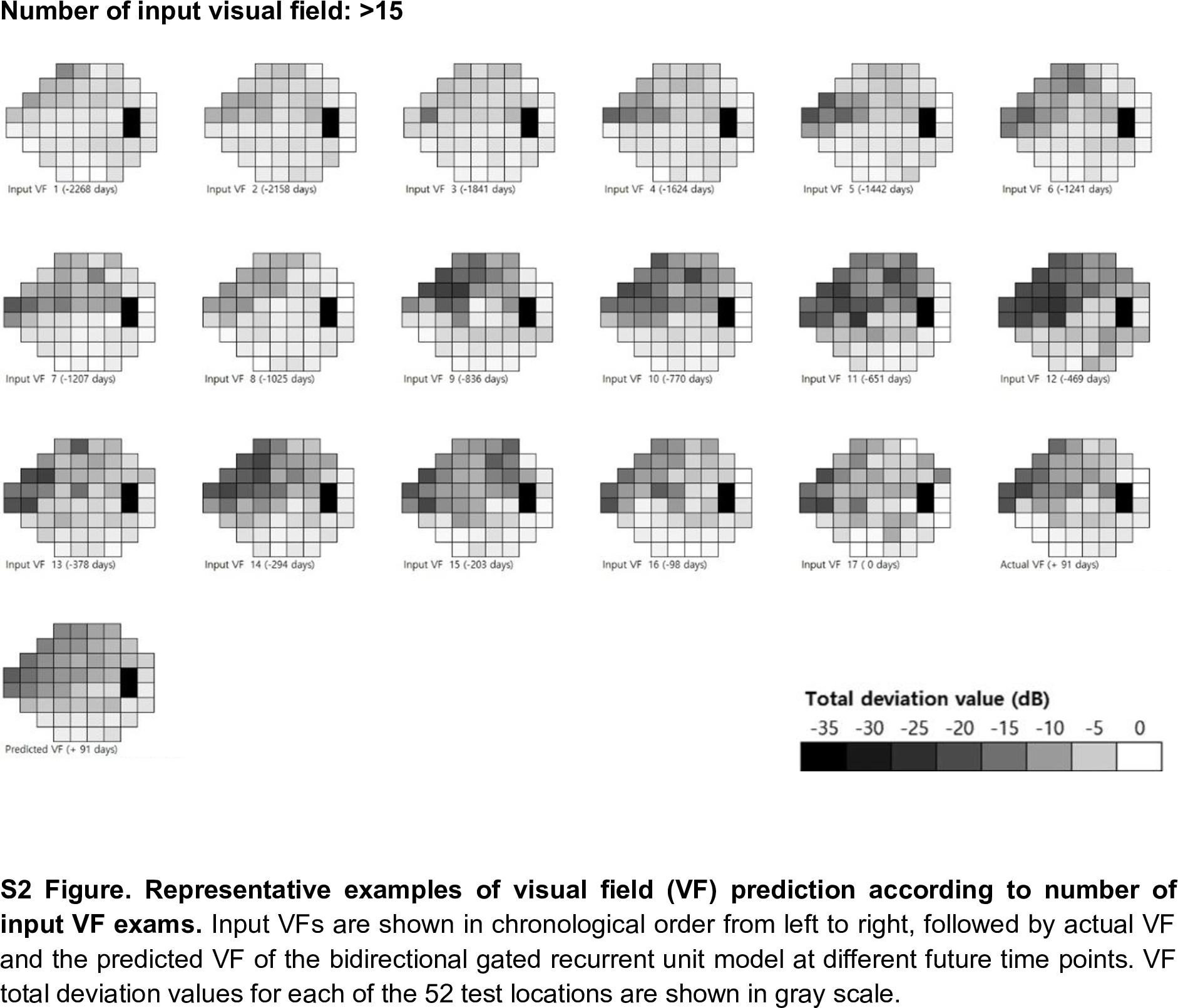

Supplement: S2 Fig — Input VFs are shown in chronological order from left to right, followed by actual VF and the predicted VF of the bidirectional gated recurrent unit model at different future time points. VF total deviation values for each of the 52 test locations are shown in gray scale. (TIF) [file pone.0307498.s002.tif]

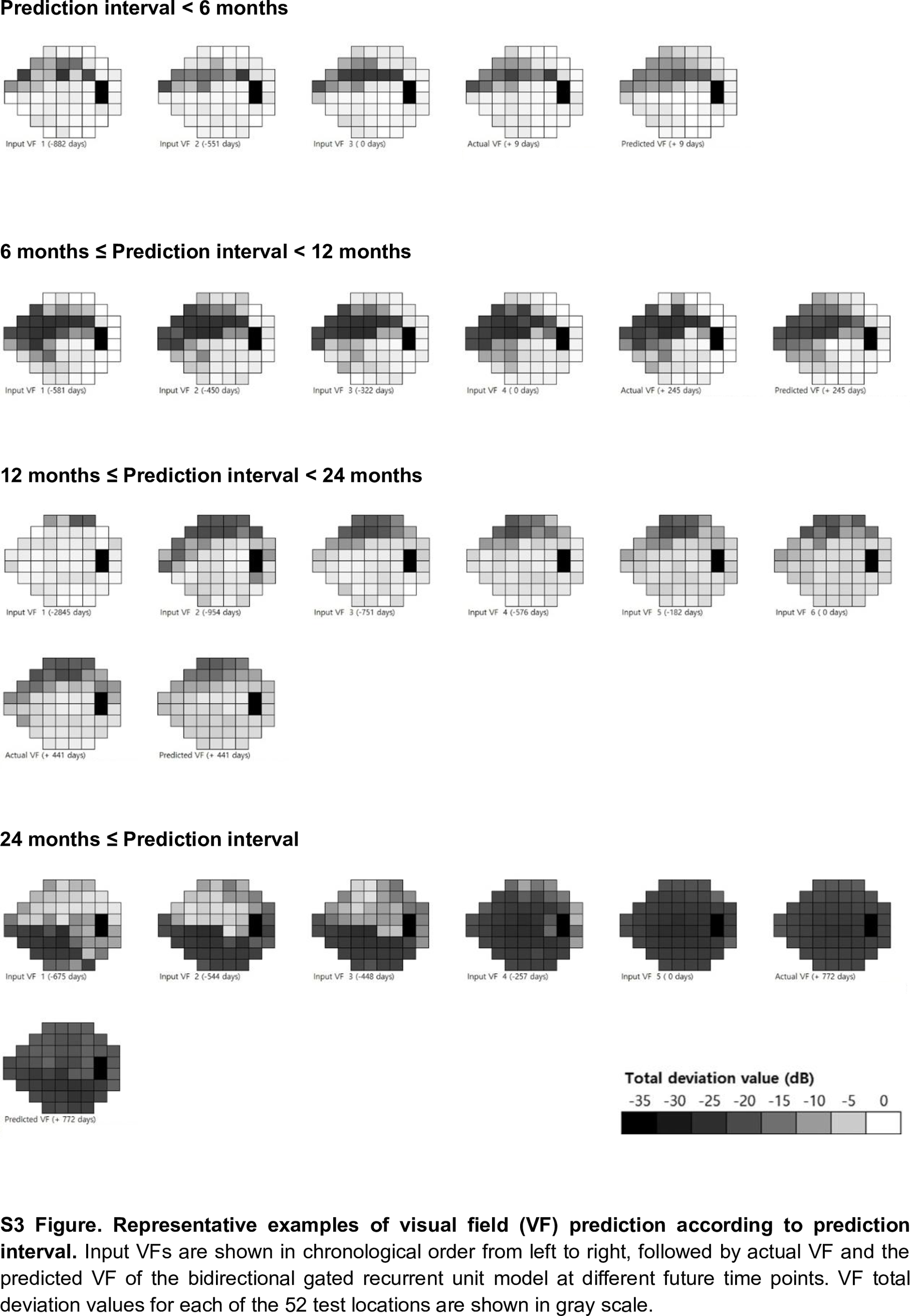

Supplement: S3 Fig — Input VFs are shown in chronological order from left to right, followed by actual VF and the predicted VF of the bidirectional gated recurrent unit model at different future time points. VF total deviation values for each of the 52 test locations are shown in gray scale. (TIF) [file pone.0307498.s003.tif]
