# Supplementary material for: Bidirectional gated recurrent unit network model can generate future visual field with variable number of input elements
Source: PLoS One. 2024 Aug 27;19(8):e0307498. doi: 10.1371/journal.pone.0307498 (PMC11349096; doi:10.1371/journal.pone.0307498)
Supplement: Attachment — Submitted filename: Response to Reviewers.docx [file pone.0307498.s004.docx]

Date: April.21.2024

Dear, Natasha Gautam, MBBS, MS

Academic Editor

PLOS ONE

Dear, Dr. Gautam

Attached please find the revised manuscript entitled “Bidirectional gated recurrent unit network model can generate future visual field with variable number of input elements”. A point-by-point response to each of reviewer’s comments follows. The authors wish to thank the Editorial Board for their thoughtful consideration and recommendations, and hope that this revised manuscript now meets your requirements for publication.

Journal Requirements:

1. “This work was supported by the National Research Foundation of Korea grants (No. RS-2023-00247504 and by Convergence Medical Institute of Technology R&D project (CMIT2023-00), Pusan National University Hospital.” Please provide an amended statement that declares *all* the funding or sources of support (whether external or internal to your organization) received during this study, as detailed online in our guide for authors at <http://journals.plos.org/plosone/s/submit-now.>

Please also include the statement “There was no additional external funding received for this study.” in your updated Funding Statement. Please include your amended Funding Statement within your cover letter. We will change the online submission form on your behalf.

Response: We confirmed that we have described all of the funding or sources of support received during this study. We added the following sentences in our updated Funding Statement as shown below.

“There was no additional external funding received for this study. The funders had no role in study design, data collection and analysis, decision to publish, or preparation of the manuscript.” We have included the amended Funding Statement within our cover letter accordingly.

2. Please include captions for your Supporting Information files at the end of your manuscript, and update any in-text citations to match accordingly. Please see our Supporting

Response: We have included a caption for the supplemental figures at the end of the manuscript and updated the in-text citations.

3. This is a well written manuscript. I would advise to move the results and details of training dataset including table 1 from methodology to results section, before describing the results about test dataset. Similarly, the authors described the criteria to determine glaucoma severity (early, moderate and severe) in results section, which should be written in methodology instead of results.

Response: Thank you for the comment. We moved the results and details of the training dataset including Table 1 from the methodology to the results section before describing the results about the test dataset. We moved the criteria for glaucoma severity (early, moderate and advanced stage) from the results section to the methods section.

Reviewer’s comment

1. Recurrent neural networks (RNNs) are well-established deep learning (DL) techniques for handling sequential data. However, recent advances have led to the development of newer methods (e.g., transformers) that may provide surprisingly good results. I believe that adding comparisons with a standard (basic) and state-of-the-art method to the test datasets could further validate the results and increase the manuscript's contribution to the field.

Response: Thank you for the comment. In fact, we have already tested “transformer network” as well as BiGRU model. As you mentioned, “transformer network” is a SOTA algorithm and we were very excited to use it. We expected its overwhelming performance just like chatBot does but on the contrary to our expectation, the performance was not so good. Now we are re-trying transformer network to solve the issue. We are also considering to use GTN (Gated Transformer Network) because visual field data contains channel like data (i.e. TDV, PDV, THV). Other than transformer network, we are trying another new architecture, CRU (Correlation Recurrent Units: A Novel Neural Architecture for Improving the Predictive Performance of Time-Series Data, <https://ieeexplore.ieee.org/abstract/document/10264112>). CRU internally utilizes correlation between input data similar to attention method in transformer. We expect CRU is lighter than transformer but it will show better performance than GRU. Taken together all above methods, we are planning to write a next paper comparing various SOTA architectures including transformer. At the moment, research is still ongoing and we did not yet conclude anything. We are very much thankful of your valuable suggestion but we would be glad if you can wait us to complete the research.

2. With such a large number of VF tests, I wonder how the authors digitally access the values in the VF reports. Meanwhile, what are the quality control steps to ensure that the digitally obtained values are the same as those in the HVF reports?

Response: Thank you for the comment. To obtain such a large amount of visual field data digitally, Zeiss provides a “study mode” in their HVF machine. To activate this mode, you need license key from Zeiss. When study mode activated, you can export all exam data as “XML” files (1 exam per 1 XML file). We developed a custom software to transform XML files to a single excel file which contains all patients’ exam data. Because the manufacturer, Zeiss, guarantees XML file data exported from HVF machine is exactly same as HVF reports, we do not need quality control step. We also confirmed that our custom software converts XML file to excel file without error.

3. The manuscript provides a solid quantitative analysis. Including qualitative results for a few best and worst cases in each prediction category (i.e., glaucoma severity, number of VF tests, and prediction intervals) would substantially provide valuable insights and enrich the narrative and depth of the study.

Response: Thank you for the comment. We have added qualitative results for a representative example for different glaucoma severity, number of input visual field exams, and prediction interval as supplementary materials (S1_fig, S2_fig, S3_fig.pdf including captions and legends) shown as below. and updated the in-text citations.

**Early stage**

**Moderate stage**

**Advanced stage**

**S1 Figure. Representative examples of visual field (VF) prediction according to glaucoma severity (early, moderate and advanced) based on patient’s last VF mean deviation (MD) value**. Input VFs are shown in chronological order from left to right, followed by actual VF and the predicted VF of the bidirectional gated recurrent unit model at different future time points. VF total deviation values for each of the 52 test locations are shown in gray scale.

**Number of input visual field: 3~5**

**Number of input visual field: 6~10**

**Number of input visual field: 11~15**

**Number of input visual field: >15**

**S2 Figure. Representative examples of visual field (VF) prediction according to number of input VF exams.** Input VFs are shown in chronological order from left to right, followed by actual VF and the predicted VF of the bidirectional gated recurrent unit model at different future time points. VF total deviation values for each of the 52 test locations are shown in gray scale.

**Prediction interval < 6 months**

**6 months ≤ Prediction interval < 12 months**

**12 months ≤ Prediction interval < 24 months**

**24 months ≤ Prediction interval**

**S3 Figure. Representative examples of visual field (VF) prediction according to prediction interval.** Input VFs are shown in chronological order from left to right, followed by actual VF and the predicted VF of the bidirectional gated recurrent unit model at different future time points. VF total deviation values for each of the 52 test locations are shown in gray scale.

4. The manuscript demonstrates a thorough analysis using an Asian dataset, which provides valuable insights specific to this population. To enhance the clarity and applicability of the manuscript, I believe it would be beneficial to acknowledge that the focus on this particular dataset may influence the generalizability of the findings to other racial groups. Including a brief discussion of this aspect could further strengthen the study by highlighting areas for future research and the potential application of a more diverse dataset.

Response: Thank you for the comment. We have added data bias and generalization issues due to the single ethnic group in the Asian dataset to the limitation section. We have added following sentences as shown below.

“Lastly, in this study, the RNN algorithm was trained on a single ethnic group consisting of Asian, which can lead to data bias (racial bias). To reduce this issue and ensure data generalization, it is crucial to build a diverse and representative training datasets of all races and ethnicities to test and report its performance.”

Sincerely,

Jiwoong Lee, MD, PhD

Department of Ophthalmology, Pusan National University Hospital, 179 Gudeok-ro, Seo-gu, Busan 49241, South Korea

Tel: +82-51-240-7326

Fax: +82-51-242-7341

E-mail: alertlee@naver.com
